# BCG Moreau Polish Substrain Infections in Patients With Inborn Errors of Immunity: 40 Years of Experience in the Department of Immunology, Children's Memorial Health Institute, Warsaw

**DOI:** 10.3389/fped.2022.839111

**Published:** 2022-05-19

**Authors:** Ewa Bernatowska, Małgorzata Pac, Edyta Heropolitańska-Pliszka, Barbara Pietrucha, Nel Dąbrowska-Leonik, Małgorzata Skomska-Pawliszak, Katarzyna Bernat-Sitarz, Katarzyna Krzysztopa-Grzybowska, Beata Wolska-Kuśnierz, Nadia Bohynikova, Ewa Augustynowicz, Ewa Augustynowicz-Kopeć, Maria Korzeniewska-Koseła, Anna Wieteska-Klimczak, Janusz Książyk, Teresa Jackowska, Mirjam van den Burg, Jean-Laurent Casanova, Capucine Picard, Bożena Mikołuć

**Affiliations:** ^1^Department of Immunology, Children's Memorial Health Institute, Warsaw, Poland; ^2^Department of Sera and Vaccines Evaluation, National Institute of Public Health – National Institute of Hygiene, Warsaw, Poland; ^3^Department of Epidemiology, National Institute of Public Health – National Institute of Hygiene, Warsaw, Poland; ^4^Department of Microbiology, National Tuberculosis Reference Laboratory, National Tuberculosis and Lung Diseases Research Institute, Warsaw, Poland; ^5^Department of Tuberculosis Epidemiology and Surveillance, National Tuberculosis and Lung Diseases Research Institute, Warsaw, Poland; ^6^Department of Paediatrics, Nutrition and Metabolic Diseases, Children's Memorial Health Institute, Warsaw, Poland; ^7^Department of Paediatrics, Medical Centre of Postgraduate Education, Warsaw, Poland; ^8^Department of Paediatrics, Bielanski Hospital, Warsaw, Poland; ^9^Department of Pediatrics, Laboratory for Pediatric Immunology, Leiden University Medical Center, Leiden, Netherlands; ^10^Howard Hughes Medical Institute, New York, NY, United States; ^11^St. Giles Laboratory of Human Genetics of Infectious Diseases, Rockefeller University Hospital, New York, NY, United States; ^12^Necker Hospital for Sick Children, Paris Descartes University, Paris, France; ^13^Laboratory of Human Genetics of Infectious Diseases, Imagine Institute, Necker Hospital for Sick Children, Paris, France; ^14^Necker Hospital and School of Medicine, University Paris Descartes, Paris, France; ^15^Imagine Institute, Université de paris, Paris, France; ^16^Study Centre for Primary Immunodeficiency, Necker-Enfants, Malades Hospital, Assistance Publique des Hôpitaux de Paris, Paris, France; ^17^Department of Paediatrics, Rheumatology, Immunology and Metabolic Bone Diseases, Medical University of Bialystok, Bialystok, Poland

**Keywords:** Inborn Errors of Immunity, BCG infection, susceptibility to BCG infection, BCG Moreau vaccine, disseminated BCG infection, NK cells

## Abstract

**Objective:**

We aimed to assess BCG (Bacillus Calmette-Guérin) complications in patients with Inborn Errors of Immunity (IEI), according to the inherited disorders and associated immunological defects, as well as the different BCG substrains.

**Material:**

We studied adverse reactions to the locally-produced BCG Moreau vaccine, analyzed in patients with IEI diagnosed between 1980 and 2020 in the Department of Immunology, Children's Memorial Health Institute (CMHI), Warsaw. These results were compared with previously published studies.

**Results:**

Significantly fewer disseminated BCG infections (BCGosis) were found in 11 of 72 (15%) SCID (Severe Combined Immunodeficiency) NK (Natural Killer)-phenotype patients, when compared with the 119 out of 349 (34%) (*p* = 0.0012) patients with SCID with BCG in other countries. Significantly fewer deaths caused by BCGosis were observed (*p* = 0.0402). A significantly higher number of hematopoietic stem cell transplantations (HSCTs) were performed in the CMHI study (*p* = 0.00001). BCGosis was found in six patients with Mendelian susceptibility to mycobacterial diseases (MSMD). Other patients with IEI prone to BCG complications, such as CGD (Chronic Granulomatous Disease), showed no case of BCGosis.

**Conclusion:**

The BCG Moreau substrain vaccine, produced in Poland since 1955, showed genetic differences with its parental Brazilian substrain together with a superior clinical safety profile in comparison with the other BCG substrains, with no BCGosis in patients with IEI other than SCID and MSMD. Our data also confirmed significantly fewer cases of BCGosis and deaths caused by BCG infection in patients with SCID with this vaccine substrain. Finally, they confirmed the protecting role of NK cells, probably *via* their production of IFN-γ.

## Introduction

The primary defects of cellular response, phagocytic function, and interferon-gamma-mediated immunity have been associated with mycobacteria complications, such as tuberculosis (TB), non-tuberculosis mycobacteria disease, and BCG infection (1–25). BCGosis in patients with IEI is estimated at ~0.06–1.56 cases per million doses of vaccine administered, occurring with different frequencies in individual diseases. High frequencies of serious outcomes in individuals, together with elevated frequency of lethal course of BCGosis, have been reported in patients highly susceptible to mycobacterial diseases IEI, such as patients with SCID, and those with Mendelian susceptibility to mycobacterial diseases (MSMD) (1–14). One of the biggest groups of patients with IEI with BCGosis was retrospectively analyzed by Casanova et al. (1–3). Today, the results concerning the most numerous study group, coming from 28 centers in 17 different countries and comprising 345 patients with SCID that were vaccinated with the BCG, have been published by Marciano et al. (22).

Patients with chronic granulomatous disease (CGD) are shown to be less prone to BCG infection than patients with SCID and MSMD. However, the incidence of BCG-associated complications is still considerable in this group (8, 15, 16, 19, 20). Other, rare diseases, showed susceptibility to mycobacterial complications in single cases of BCG infection (1, 8, 14, 21, 24, 25).

In Poland, the first immunizations against tuberculosis were performed in 1926. Initially, newborns were vaccinated orally, and at that time numerous adverse events were reported in the form of lymphadenitis. In 1955, the highly reactogenic BCG Danish vaccine was replaced in Poland by a locally-produced BCG Moreau vaccine, a descendant of the Brazilian BCG Moreau substrain, with a great reduction in complications. In a trial reported by Zapaśnik-Kobierska and Stopnicka, the Moreau substrain was regarded as the safest, inducing suppurative lymphadenitis in only 0.3% of the children compared with 2.4 and 4.9% in those immunized with vaccines produced with Danish and French substrains, respectively, (26). According to the study carried out several years later, 2.9% purulent lymphadenitis was observed in newborns after administration of the vaccine with the French substrain. No such complications occurred after administering a vaccine with the Moreau substrain (27). The frequency of adverse events following BCG vaccination in Poland in 1994-2000 and 2001-2010 was around 0.2 and 0.6‰, respectively. Mostly it appeared in the form of local lesions at injection sites and regional lymphadenopathy (28). In Poland, the production of the vaccine using the BCG Moreau substrain has continued to this day. The aim of the present study was to estimate the frequency of BCG complications in patients with IEI, those vaccinated at birth, and those hospitalized at the Department of Immunology, CMHI in Warsaw over a period of 40 years.

## Materials and Methods

As many as 1,822 patients were diagnosed with IEI in the Department of Immunology, CMHI, Warsaw between 1980 and 2020; about half of them are citizens of the Mazovia district, and others come from the rest of the county. In total, 200 of the patients were classified as having high susceptibility to BCG infection, and those with selected defects affecting either innate or adaptive immunity in whom BCG complications could occurr. Among them, 180 were vaccinated at birth with the BCGMoreau vaccine produced by “BIOMED-LUBLIN,” (Table 1) (29). BCGosis was diagnosed based on clinical, microbiological, and histopathological findings in compliance with the ESID diagnostic criteria (6) (https://esid.org/Working-Parties/Registry-Working-Party/Diagnosis-criteria). BCGitis referred to as a lesion inside the inoculation site (>10 mm) and/or lymphadenitis limited to the region of the inoculation site, whereas BCGosis was defined as a persistent process affecting two or more sites apart from the inoculation site. The molecular analysis of the Mycobacterium tuberculosis complex was conducted using the PCR (MTD Gen-Probe) test and the culture of mycobacterium was performed in the BACTEC 460 Tb or MGIT 960 system. Mutation analysis in patients with IEI was performed at the Department of Medical Genetics, CMHI in Warsaw, the Department of Immunology, Erasmus MC in Rotterdam, Netherlands, the Center for the Study of Primary Immunodeficiencies, Assistance Publique, Hopitaux de Paris, Necker Hospital, 75015 Paris, France, and at the Department of Pediatrics, Oncology, Hematology and Diabetology, Medical University of Łódz. Absolute numbers and percentages of circulating B and T subsets CD19/CD20, CD3, and natural killer cells (NK-CD56/CD16) were assessed and compared with reference values established in the age-matched groups of healthy children (30).

**Table 1:** BCG and other mycobacterial complications in PID patients susceptible to BCG infection hospitalized at CMHI.

**PID diagnosed**	**BCG vaccinated patients 180/200**	**Gene defect**	**TB**	BCGitis^*^	**BCGosis**	**Positive PCR/culture**	**Death from BCGosis**
SCID	72/85	9 subtypes See Figure 1	1	18	11	9/6	3
MHC class II deficiency	1/1	*RFXANK*	-	-	-	-	-
ZAP-70 deficiency	1/1	*ZAP70*	-	-	-	-	-
CD40 ligand (CD154) deficiency	6/8	*CD40LG*	-	-	-	-	-
IFN-γ receptor 1 deficiency (MSMD)	3/3	*IFNGR1*(*n* = 2)	-	3	3	2/2	0
IL-12p40 deficiency (MSMD)	3/3	*IL-12Rβ*	1	3	3	2/2	1
XL CGD	38/38	*CYBB* (*n* = 29) mutation	8	3	-	-	-
AR CGD	25/28	*NCF1* (*n* = 20) and *CYBA* (*n* = 1)	4	1	-	-	-
HIES	20/20	LOF mutation in *STAT3* (*n* = 15)			-	-	-
STAT1 GOF	2/2	GOF mutations in STAT1	1	1	-	-	-
EDA-ID due to NEMO/IKBKG deficiency	2/2	*IKBKG*	1		-	-	-
Activated p110δ syndrome (APDS2)	2/2	*PIK3R1*	-	-	-	-	
GATA2 deficiency	1/1	*GATA2*	-	-	-	-	-
DiGeorge/velocardio-facial syndrome (phenotype T-B + NK+)	2/4	22q11 del	-	1	-	-	-
CHARGE syndrome (phenotype T-B + NK+)	2/2	*CHD7*	-	1	-	-	-

* *Significantly fewer BCGitis complications in PID patients prone to BCG infection (in 6 out of 102), in comparison with SCID and MSMD patients together (in 24 out of 78) (p < 0.00001)*.

Statistical analyses were performed using STATISTICA v 10.0 and Microsoft Excel v 2007. Quantitative variables were characterized by the arithmetic mean of standard deviation or median or max/min. Qualitative variables were presented as counts and percentages. To check if a quantitative variable derived from the population of normal distribution, the Shapiro–Wilk test was used. Statistical significance of differences between groups (model of unpaired variables) was processed with the Student's *t*-test or Mann–Whitney *U*-test. The Chi-squared test and Fisher's exact test for independence were used for qualitative variables. To determine dependence, strength, and direction between variables, correlation analysis was applied by determining Pearson or Spearman's correlation coefficients. In all calculations, a statistical significance level of *p* = 0.05 was used. The statistical analysis of the retrospective study of 72 patients with SCID hospitalized at the CMHI was compared with that of 349 patients with SCID analyzed by Marciano et al. (22).

### Ethics Statement

This study was approved by the Bioethics Committee of the CMHI - Resolution No. 5/KBE/2020 on 23 January 2020.

## Results

**Figure 1:**
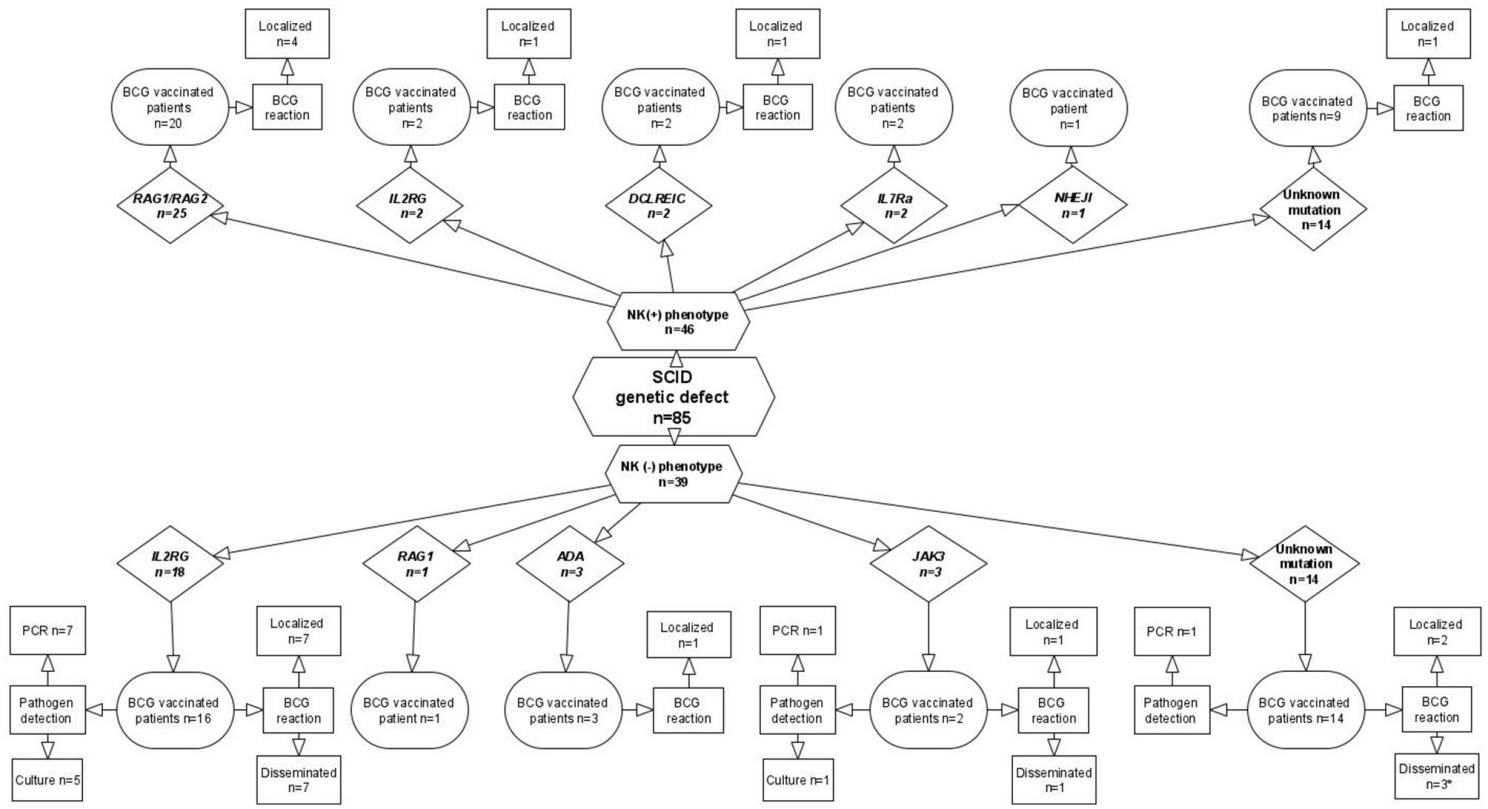
SCID diagnosis and BCG-associated complications in patients hospitalized at CMHI.

In total, 85 patients were diagnosed with SCID. A pool of 72 patients was vaccinated with the BCG at birth. Within this group, BCGosis occurred in 11 patients with SCID, and BCGitis was present in 18 patients (Table 1; Figure 1). Age at onset of BCGitis and BCGosis reaction occurred between 1 and 7 months of age. BCGosis was diagnosed only in patients with SCID with the NK-phenotype; in 7 out of 18 patients with the *IL2RG* mutation, in 1 out of 3 with JAK3 (Janus Kinase 3) deficiency, and in 3 individuals with unknown mutations. BCGosis was not present in the group of patients with NK+ SCID (Figure 1).

The clinical outcome of BCGosis in 10 patients with SCID diagnosed before 2015 has been described in previous publications (6, 23). Between 2015 and 2019, only 1 patient with an *IL2RG* mutation (Interleukin 2 receptor gamma), out of 20 newly recognized patients with SCID, developed BCGosis. This patient had BCG meningitis, bronchopneumonia, and osteomyelitis with pathological fractures. Long-lasting, anti-TB therapy with 4-drugs was interrupted twice during a period of 16 months after HSCT. However, BCGosis was cured, and the therapy was discontinued after 22 months.

BCGosis in 11 (15%) patients with SCID hospitalized at the CMHI constitutes a significantly lower number compared with the 119 (34%) in the Marciano study (*p* = 0.0012). However, in 18 (25%) patients with SCID with localized BCG infection (BCGitis), the rate was insignificantly higher compared with the 60 in the Marciano study (17%) (*p* = 0.1341) (Table 2). Mortality caused by BCGosis complications in the Marciano cohort of patients with SCID was significantly higher: 46 (13%) deaths out of 349 (*p* = 0.0402). In the CMHI cohort, three (5%) deaths occurred before 1985 (6). BCGosis complications in the CMHI study were found only in 11 out of 72 patients with SCID with a low number of NK cells (*p* < 0.000001), while localized complications were observed in both groups, which were insignificantly more common in patients with NK-SCID (*p* = 0.5987) (Table 3). Dual drug anti-TB therapy (isoniazid and rifampicin) was administered to 18 patients with SCID and 2 patients with complete di George syndrome (cDGS), all with BCGitis. In 8 out of 11 patients with SCID, BCGosis was successfully treated with 4 or 5 anti-TB drugs (isoniazid, rifampicin, ethambutol, ciprofloxacin, and aminoglycoside). HSCT was administered in 61 patients (85%) in total vs. 190 (54%) patients with SCID in the Marciano study, the number being significantly higher in the CMHI study (*p* < 0.00001) (Table 4). A total of 58 out of 349 patients with SCID were diagnosed in Brazil and constitute the largest cohort of this study (22). Similarly, fewer HSCT procedures were performed in patients with SCID in Brazil with a total of 24 (46%) patients compared to the 61 patients at the CMIH (85%) (*p* < 0.00001). BCGitis and BCGosis complications within our group of patients with SCID developed in 29 (40%) patients in comparison to the 177 (51%) individuals in the Marciano study (22).

**Table 2:** Comparison of BCG-associated complications in two study groups.

**SCID patients**	**CMHI study**	**Marciano study (22)**	***P*-value**
	***n* = 72**	***n* = 349**	
Disseminated	11 (15%)	119 (34%)	0.0012
Localized	18 (25%)	60 (17%)	0.1341
Mortality	3 (4,2%)	46 (13%)	0.0402

**Table 3:** Number of NK cells in 72 patients with SCID vaccinated with the BCG-vaccine hospitalized at CMHI.

**SCID**	**NK cells μl (normal range > 200 μl)**	**Localized**	**Disseminated**
NK – SCID (*n* = 36)	46,5 (2–190)	11 (55%)	11 (100%)
NK + SCID (*n* = 36)	398 (203–3771)	9 (45%)	0 (0%)
*P-*value	*P* <0.000001	*P* = 0.5987	*P* = 0.00031

**Table 4:** Composition of the frequency of HSCT procedure implementation in patietns with SCID.

	**CMHI**	**Marciano study (22)**	**Brazil study (22)**	***P-*value**
	**(study 72)**	**(*n* = 349)**	**(*n* = 58)**	
HSCT	61 (85%)	190 (54%)		*P* <0.00001
	61 (85%)		24 (46%)	*P* <0.00001

BCGosis and BCGitis occurred in a total of six patients with MSMD; in three patients with IL-12 and IL-23 receptor β1 chain deficiency; in two patients with partial autosomal dominant(AD) defect of IFNGR1; and in one patient with IFNGR deficiency diagnosed by flow cytometry but not confirmed genetically. Additionally, in two patients with INFGR (Interferon γ Receptor 1 deficiency), a partial AD defect, the *Mycobacterium avium complex* disseminated infection was observed (Table 1). The fatal course of infection occurred in an infant with partial IFNGR1 (Interferon γ Receptor 1 deficiency), in whom the cause of death was CMV(Cytomegalovirus) infection, and in one infant with IL12R (Interleukin 12 subunit 40) deficiency in whom anti-TB therapy was stopped. In the remaining four patients, the BCG infection was cured and none of them showed a relapse of the BCG infection. They are now 5, 6, 10, and 20 years old, respectively.

CGD has been recognized in 66 patients. For 23 patients (24%) out of the 66 diagnosed in the early 1980s, no genetic diagnostics were performed (Table 1). A total of 29 and 20 patients were diagnosed with the XL (X-linked) and AR(autosomal recessive) forms of CGD, respectively, based on family history as well as on the clinical and biochemical phenotype of the disease (https://esid.org/Working-Parties/Registry-Working-Party/Diagnosis-criteria). BCGitis was noted in four patients. Lymphadenopathy was observed in 37 patients with 85 episodes of suppurative lymphadenitis. There were 68 surgical interventions performed, but this was without a histopathological examination of lymph nodes. *Staphylococcus aureus* infection was regarded as the most common cause. *Mycobacterium tuberculosis* was not regarded as a causative agent. Before 1980, 12 patients with lung tuberculosis were treated in district hospitals. Those patients had possible tuberculosis, diagnosed on clinical criteria (31). Since 1980, there have not been any tuberculosis cases in our CGD patients.

The group of other patients with IEI prone to BCG infection is shown numerically in Table 1. None of these patients developed BCGosis, and the number of BCGisis cases in this group was significantly lower (*n* = 0, 000001) than in the 78 patients with SCID and MSDM, who developed a total of 24 localized reactions compared to 102 other patients with IEI, in whom only six cases of BCGitis were noted (Table 1). The percentage of vaccinated Polish newborns was high, about 90%, for years. Six of our patients with SCID were not vaccinated with BCG because of positive family history.

## Discussion

In Poland, the incidence of tuberculosis has been gradually decreasing for years, with 14.3 cases per 100,000 population in 2018, but the criteria to end mass vaccination have still not been met (32, 33). Since 2006, according to WHO recommendations, the BCG vaccine in Poland is currently administered just once at birth (34). The percentage of vaccinated Polish newborns is high, about 90%, and has been for years. Six of our patients with SCID were not vaccinated with BCG because of positive family history.

There are a significantly smaller number of BCGosis cases in patients with SCID due to the BCGMoreau vaccine in comparison to the number of this complication in patients vaccinated with BCG vaccines with other substrains, and this has been observed over 30 years at the CMHI. An insignificantly higher number of BCGitis cases occurred. Both BCGitis and BCGosis complications occurred in 40% of our patients with SCID in comparison with the 51% of cases in the Marciano study (22). None of the other patients with IEI presented with BCGosis in our study (Table 1). This observation once more confirms the safety profile of the locally produced BCG Moreau vaccine (23).

A French national retrospective study carried out from 1974 to 1994 reported the clinical outcome of BCG complications in 16 patients with no confirmed immunodeficiency; 8 out of 16 patients vaccinated with the BCG Pasteur substrain and 2 other patients vaccinated with the Glaxo and Danish substrains developed BCGosis and died as a result (2). In Canada, four infants with SCID and one with HIV (Human Immunodeficiency Virus) vaccinated at birth with the BCG Pasteur Merieux Connaught vaccine also developed BCGosis and subsequently died (17). Another retrospective study conducted between 2007 and 2012 in China revealed the clinical outcome of PID in patients vaccinated at birth with vaccines produced locally by four different manufacturers with the use of BCG Danish substrain (8). A group of 14 out of 74 patients in this study, including 32 well-defined patients with IEI, developed BCGosis, while 2 patients with SCID died. In the Czech Republic, 9 out of 12 patients with SCID vaccinated at birth with a locally-produced BCG Danish substrain vaccine developed BCGosis, with 5 of them subsequently dying (18). The biggest study summarized data from 17 countries and examined the incidence of BCGosis in 349 patients with SCID (22). This study shows no significant differences between the reactogenicity of individual substrains; however, the Danish substrain caused more BCGosis compared to other substrains. In our recently published study, significantly fewer BCGosis cases occurred in our 52 patients with SCID hospitalized at the CMHI between the years 1980 and 2015 compared to the incidence of BCGosis in 349 patients with SCID vaccinated with other BCG substrains (22, 23). In this study, BCGosis occurred only in patients with SCID with the T-B+ NK- phenotype. In that study group, the number of NK cells was statistically higher, thus providing essential protection against BCGosis for patients with SCID. Since 2016, there have been 20 newly diagnosed patients with SCID, and only in 1 patient was BCGosis found. The patient had SCID with the T-B + NK-SCID phenotype and with the *IL2RG* mutation. This finding proves the hypothesis that NK cells can provide the primary source of IFN-γ production during blood exposure to the Mycobacterium bovis BCG substrain (35–37). The crucial role of NK cells in protection against mycobacterial infection has been documented once more. Recently, one of the biggest studies of 82 RAG1/2 SCID with phenotype NK+ was published (38). TwIn total, 22 of the patients had been vaccinated with BCG: Moscow-368 substrain, BCG-Prague originally from the Danish substrain, or BCG Sofia SL 222. Four of them developed BCGitis, and one BCGosis caused by the BCG Sofia SL 222 vaccine (38). Generally, none of our 29 patients with SCID, included in this study, vaccinated with the BCG Moreau vaccine developed BCGosis. In a recently published review, which summarized BCG complications in patients with PID, BCG infection was documented in 78 patients with SCID with the NK- SCID phenotype and in 40 with the NK+ phenotype, in whom BCG complications are probably related to factors other than Moreau BCG substrains (14).

MSMD is at equal risk of BCG complications to patients with SCID. All mutations in several gene loci detected for MSMD lead to a defect in IFN-γ or IL12/23 (Interleukin 12/23) receptors or in signal transduction pathways, which impairs the response to mycobacterial infections (8, 13, 14). A total of six patients with MSMD developed BCGosis, and none of the four treated with the anti-TB drug showed a relapse of the BCG infection compared to the fatal outcome in some patients vaccinated with the BCG vaccine based on different substrains (mostly of Danish origin) (8–10, 13, 14).

CGD is the largest group of patients less prone to BCG complications. However, BCGosis and fatal outcomes of this infection were reported in individual patients (9–11%) (8, 13–16, 20, 24). CGD is characterized by impaired production of reactive oxygen species and mutations in one of the following genes *CYBB, CYBA, NCF1, NCF2, NCF4, CYBC1/EROS*, which are the causes of the low levels of H_2_O_2_ production cells necessary for protection against mycobacterial infections (4, 15, 20). Clinical profiles of our 66 patients with CGD were documented over the years (5, 15). The clinical outcome did not show any form of BCGosis. None of the 37 patients with recurrent episodes of lymphadenitis were considered to be a cases of BCG infection and were treated with anti-TB drugs. The biggest study assessing clinical outcomes looked at 446 patients with CGD originating from 16 European and Arabic/North African countries and was published in 2009 (15). BCG complications occurred in 34 out of 429 patients (8%) and included a high number of French patients (22 = 65%) vaccinated with the BCG Danish substrain vaccine. Among the group of 30 Polish patients with CGD included in the study, none of them presented with BCGosis. The aforementioned findings are in contradiction with a wide range of publications reporting BCG complications in patients with CGD worldwide caused by BCG Danish or Pasteur substrains (8, 13–16, 20, 24). A group of 23 Chinese patients with CGD vaccinated with the BCG Danish vaccine developed BCGosis despite anti-TB treatment, and 3 of them died (8). In a recent study of 71 patients with CGD originating from 20 different countries (Polish patients were not included), BCGitis was noted in 23 cases and BCGosis occurred in 8 individuals, 3 of whom died (20). It should be stressed that our 29 patients with XL CGD with a mutation in CYBB, encoding Rgp91 ^*phox*^, are extremely sensitive to mycobacterial infection in their lives because of the mutation, which has been classified under the category of MSMD (20). This is confirmed by a systematical review of BCG complications in patients with IEI, which shows that patients with CGD and CYBB gene defects presented BCG complications more often than patients with other mutations (14).

XL-HIGM syndrome (CD40 ligand (CD154) deficiency), which is caused by mutations in the CD40 Ligand (*CD40LG*) impairing T-cells activity, may lead to mycobacterial infections in patients (8, 13, 14). Two Chinese patients with HIGM (hyper-IgM syndrome) vaccinated with the BCG Danish substrain vaccine developed BCGosis, which was not cured for over 2 years (8).

Cristiane Nunes-Santos et al. published a systematic review of BCG complications in patients with IEI with selected defects affecting either innate or adaptive immunity published after 2010 (21). BCGosis was reported in 4 (8%) out of 19 patients with a gain-of-function STAT1 (signal transducer and activator of transcription 1) mutation, impairment of IFN -γ responses immunity to mycobacterial infection, and in 1 out of 357 patients with GATA2 deficiency, a deficiency in hematopoietic transcription factor GATA2 (21, 39, 40). In a large group of our HIES (hyper-IgE syndrome) patients, reported by Nunes-Santos et al., no BCG complications were noted (21). Nevertheless, there are individual reports of BCGosis, with fatal course of infection, in patients vaccinated with the Danish substrain (8, 14, 41). Among other rare diseases prone to BCG infections, single BCG complications were noted in patients with EDA-ID due to NEMO/IKBKG deficiencies and those with *IKBKG* mutations and impaired NK cell cytotoxicity. BCG infection was occurred also in patients with activated PIK3Kδ syndrome 2 caused by a heterozygous splice site mutation in *PIK3R1 (Phosphoinositide-3-Kinase Regulatory Subunit 1)* (13, 42–44). However, it should be stressed that the number of patients vaccinated with BCG might not have been sufficient to consider that the frequency of BCG complications is low.

Patients with Di George syndrome are generally regarded to be less prone to mycobacteria infections, however, those with complete Di George syndrome or CHARGE syndrome with phenotype T-B+NK+, theoretically, should be more prone to BCG infection compared to patients with SCID. Two cases of BCGosis in patients with complete DGS have been reported in the literature so far (1, 14).

In 1924, the prevalence of the BCG substrain began. It is now known that different BCG substrains vary from the parental BCG obtained in 1921 as well as due to deletions, duplications, and accumulation of SNP mutations. These differences may constitute one of the causes of the observed differences in immunogenicity or residual virulence of particular BCG substrains; nevertheless, whether and how these genetic differences affect BCG efficacy remain largely unknown (45–47). Recent studies showed that 188 T-cell epitopes essential to the human immune response to the Mycobacterium tuberculosis infection were lost in BCG strains to different degrees, however, it was emphasized that it is not the number of epitopes but the type that is most important (48). It is essential to point out bacterial genetics plays an important role in determining the ability of BCG substrains to prevent TB morbidity. Still, an efficient TB vaccine program should also take into account other factors such as minimizing adverse reactions to BCG vaccination and potential variable susceptibility to mycobacterial chemotherapeutics by different BCG substrains (49).

Compared to other BCG strains, BCG-Japan, BCG-Moreau, and BCG-Glaxo are defective in the production of phthiocerol dimycocerosates (PDIMs) and phenolic glycolipids (PGLs)—two cell wall lipids that are regarded as important for the virulence of *Mycobacterium tuberculosis* and *Mycobacterium bovis*, suggesting that these BCG strains are more attenuated than others and manifest a smaller number of complications following vaccination in children (50, 51). In BCG-Moreau, the PDIM and PGL defects are due to the 975-bp deletion of the distal end of *fadD26* and the start of *ppsA* (52). BCG strains exhibit many genetic polymorphisms in the phoP-phoR locus, i.e., the system known to play a role in virulence. BCG-Russia, BCG-Japan, and BCG-Moreau have an IS6110 insertion in the promoter region of PhoP, which may eliminate the auto-repression regulatory mechanism of this system (52, 53). Another polymorphism revealed only in the BCG-Moreau genome is a deletion within *Rv3887c*. This gene encodes a membrane transport protein that is part of the ESX-2 type VII secretion system and might influence the immunogenicity of the vaccine strain (52, 54).

We did not observe this deletion in the BCG-Moreau substrain used in Poland, therefore this region had to be removed after 1954 in the BCG-Moreau genome used in Brazil (55). The clinical outcome of BCG complications in patients with SCID vaccinated with the “mother” or the “daughter” Moreau substrain confirmed genetic differences in these two substrains (7, 23). A group of 29 of 60 (48.3%) patients with SCID immunized in Brazil with the Rio de Janeiro substrain developed BCGosis (7). In contrast, in our 52 patients with SCID vaccinated with the locally-produced BCGMoreau vaccine, 10 people (19%) developed BCGosis (23). In the present study, differences are more visible: after the next 4 years, BCGosis occurred in 11 (15%) of 72 patients diagnosed with SCID in comparison with the Brazilian vaccinated population (7).

Another very important mutation characteristic of the BCG-Moreau strain is a deletion of 7608 bp (RD16) that results in the truncation of a putative TetR transcriptional regulator, i.e., the ortholog of M. tuberculosis rv3405c (56). Some studies draw attention to the fact that “early” substrains such as BCG-Moreau are considered more immunogenic in contrast to substrains obtained after 1927, as the latter does not contain RD2 and there is no point mutation at mma3, which is responsible for impaired methoxymycolate production (57, 58).

Generally, given the residual virulence of BCG substrains, two groups to be distinguished are the more virulent ones represented by BCG-Russia, BCG-Sweden, BCG-Danish, and BCG-Pasteur, and the less virulent group, including BCG-Japan, BCG-Moreau, BCG-Glaxo, and BCG-Prague. This division can now be explained at the molecular level and can be associated with the action of many mutations in well-recognized virulence factors (e.g., ESX-1, PDIM/PGL, phoP) (59). It should be emphasized that there is no evidence so far that vaccine efficacy was associated with BCG strain (60).

In summary, the propensity for adverse effects for the different BCG substrains is undoubtedly related to their genetic differences. The BCG Moreau, the locally-produced vaccine, is considered safe, and this can be explained at molecular levels in the Moreau substrain, including a mutation that causes a lack of PDIMs and PGLs production—lipids with a known role in the *Mycobacterium tuberculosis* and *Mycobacterium bovis* virulence (50–55).

The strain used in Poland since 1955 for BCG vaccine production is the origin of the Brazilian BCG Moreau substrain, but they differ genetically in at least one region, which may explain the observed superior clinical safety profile of the Polish strain in comparison to the Rio de Janeiro parent BCG substrain and the Danish and Pasteur substrains.

This paper confirms previous findings indicating a protective role of NK cells in BCGosis, which is also confirmed by Sharapova et al. in a large group of 82 patients with RAG1/2 SCID with the NK+ SCID phenotype, revealing fewer BCGosis complications in NK+ SCID (23, 38). A recently published systematic review of BCG complications in patients with IEI reports twice as many cases of BCG infections in SCID with NK- phenotype (14). This appears to confirm the protective role of NK cells, probably via their production of IFN-γ. However, further studies argue a lack of or a significant deficiency in NK cells play decisive roles in the protection against BCGosis are necessary. An equally important reason for better SCID patient outcomes was a significantly higher number of HSCT procedures as well as anti-TB therapy provided to our patients with SCID in each BCGitis case in the inoculation site, which is a therapy that continues after HSCT. However, despite using a substantially less reactogenic BCG vaccine, a high number of HSCT procedures, and early introduction of anti-TB therapy, vaccination with the BCG vaccine should be strongly contraindicated in patients with SCID. The introduction of New Born Screening should be relevant not only to screen patients with SCID but also patients with IEI susceptible to BCG and other serious infections.

## Data Availability Statement

The datasets presented in this study can be found in online repositories. The names of the repository/repositories and accession number(s) can be found in the article/supplementary material.

## Ethics Statement

The studies involving human participants were reviewed and approved by the Bioethics Committee of the Children's Memorial Health Institute in Warsaw. Written informed consent to participate in this study was provided by the participants' legal guardian/next of kin. Written, informed consent was obtained from all individuals AND/OR their parents for the publication of any potentially identifiable images or data included in this article.

## Author Contributions

EB, MP, MS-P, and BM: conception. EB, MP, MS-P, BW-K, EH-P, BP, KB-S, ND-L, EA-K, JK, MB, J-LC, AW-K, TJ, BM, CP, NB, and MK-K: investigation. EB, MK-K, MP, MS-P, and BM: writing the manuscript. EB, MP, MS-P, BW-K, EH-P, BP, KB-S, ND-L, EA-K, JK, MB, J-LC, MK-K, EA, KK-G, and EA-K: extensive editing and revision of the manuscript. All authors contributed to the article and approved the submitted version.

## Conflict of Interest

The authors declare that the research was conducted in the absence of any commercial or financial relationships that could be construed as a potential conflict of interest.

## Publisher's Note

All claims expressed in this article are solely those of the authors and do not necessarily represent those of their affiliated organizations, or those of the publisher, the editors and the reviewers. Any product that may be evaluated in this article, or claim that may be made by its manufacturer, is not guaranteed or endorsed by the publisher.
